# Is the Frequency in Somatosensory Electrical Stimulation the* Key* Parameter in Modulating the Corticospinal Excitability of Healthy Volunteers and Stroke Patients with Spasticity?

**DOI:** 10.1155/2016/3034963

**Published:** 2016-01-06

**Authors:** Marco Antonio Cavalcanti Garcia, João Marcos Yamasaki Catunda, Marcio Nogueira de Souza, Ana Paula Fontana, Sandro Sperandei, Claudia D. Vargas

**Affiliations:** ^1^Laboratório de Instrumentação Biomédica, Programa de Engenharia Biomédica, COPPE, Universidade Federal do Rio de Janeiro, Avenida Horácio Macedo 2030, CT, Bloco H, Sala 327, Cidade Universitária, 21941-914 Ilha do Fundão, RJ, Brazil; ^2^Laboratório de Neurobiologia II, Instituto de Biofísica Carlos Chagas Filho, Universidade Federal do Rio de Janeiro, Avenida Carlos Chagas Filho 373, CCS, Bloco G, Sala G1-019, Cidade Universitária, 21941-902 Ilha do Fundão, RJ, Brazil; ^3^Departamento de Biociências da Atividade Física, Escola de Educação Física e Desportos, Universidade Federal do Rio de Janeiro, Avenida Carlos Chagas Filho 540, Cidade Universitária, 21941-599 Ilha do Fundão, RJ, Brazil; ^4^Departamento de Fisioterapia, Faculdade de Medicina, Universidade Federal do Rio de Janeiro, Avenida Carlos Chagas Filho 373, CCS, Bloco K, Sala K2-49, Cidade Universitária, 21941-902 Ilha do Fundão, RJ, Brazil; ^5^Instituto de Comunicação e Informação Científica e Tecnológica em Saúde (ICICT), Fundação Oswaldo Cruz (FIOCRUZ), Avenida Brasil 4365, 21040-900 Manguinhos, RJ, Brazil

## Abstract

Somatosensory electrical stimulation (SES) has been proposed as an approach to treat patients with sensory-motor impairment such as spasticity. However, there is still no consensus regarding which would be the adequate SES parameters to treat those deficits. Therefore, the aim of this study was to evaluate the effects of applying SES over the forearm muscles at four different frequencies of stimulation (3, 30, 150, and 300 Hz) and in two intervals of time (5′ and 30′) by means of transcranial magnetic stimulation and Hoffmann's reflex (H-reflex) in healthy volunteers (Experiments  I and II). A group of stroke patients (Experiment  III) was also preliminary evaluated to ascertain SES effects at a low frequency (3 Hz) applied for 30′ over the forearm spastic flexors muscles by measuring the wrist joint passive torque. Motor evoked potentials and the H-reflex were collected from different forearm and hand muscles immediately before and after SES and up to 5′ (Experiment  I) and 10′ (Experiments  I and II) later. None of the investigated frequencies of SES was able to operate as a* key* in switching modulatory effects in the central nervous system of healthy volunteers and stroke patients with spasticity.

## 1. Introduction

On the recent years, an approach known as somatosensory electrical stimulation (SES), which consists in applying peripheral electrical stimulation below or at the motor threshold (MT) level [1–3], has been employed to treat patients with sensory-motor impairments such as spasticity [4–8]. Although there are encouraging clinical results observed in stroke [3, 9] and spinal cord lesioned [10, 11] patients, several methodological aspects of using SES therapeutically remain unresolved [12]. Chipchase et al. [13] and Veldman et al. [14] performed systematic reviews of SES parameters upon the primary motor cortex (M1) and suggest that there is insufficient evidence to determine their effects. Additionally, there also seems to be a lack of consensus regarding the effects of SES over spinal circuits mainly due to methodological constraints, which are described in more details elsewhere [15].

Among SES underlying mechanisms, Ward [16] proposed that an increase in the frequency of stimulation upon the stimulated nerve would allow the ionic current to flow more deeply, leading to the recruitment of a larger pool of somatosensory receptors from different tissues adjacent to the SES stimulation site. Thus, distinct effects upon excitability in M1 and/or spinal circuitries would be expected depending on the average output of recruited sensory fibers. However, to our knowledge, no previous studies have compared the effects of more than two SES frequencies upon corticospinal and spinal excitability.

Therefore, the aim of this study was to evaluate by means of transcranial magnetic stimulation (TMS) and Hoffmann's reflex (H-reflex) in normal volunteers the effect of applying SES over the hand and forearm muscles at four different stimulation frequencies, conducted in two experiments. Moreover, a group of stroke patients was also preliminary evaluated to ascertain SES effects at a low frequency over the forearm spastic flexors muscles on the wrist joint passive torque.

## 2. Materials and Methods

### 2.1. Subjects

The first two experiments were performed with two groups of right-handed volunteers without neurological disorders. The first was composed of fourteen volunteers (seven males and seven females; age: 19.0–39.0 years; height: 1.54–1.92 m; body weight: 53.3–97.0 kg; Edinburgh Handedness Inventory [17]: +30.0–+90.0) and the second comprised six volunteers (five males and one female; age: 20.0–37.0 years; height: 1.54–1.92 m; body weight: 56.0–97.0 kg; Edinburgh Handedness Inventory [17]: +40.0–+95.0). The criteria of inclusion and exclusion were based on Rossi et al. [18]. The first and second groups were subjected to 5′ and 30′ SES protocols, respectively, hereafter called Experiments I and II. Both application times were arbitrarily defined.

A third group composed of five chronic stroke patients (one female and four males; age: 45–70 years; height: 1.56–1.70 m; body weight: 61.0–89.0 kg) with spasticity, all right-handed (Edinburgh Handedness Inventory [17]: before stroke [+86.67–+100.0]; after stroke [−100.0–+23.33]), was also recruited for a pilot study concerning SES effects on spastic muscles (Experiment III). Among the adopted criteria of inclusion [18], the main one was to present spasticity on the right forearm limiting wrist movements with a maximum score of “2” (“More marked increase in muscle tone through most of the ranges of motion (ROM), but affected part(s) easily moved”) based on the modified Ashworth scale [19]. Furthermore, cognition and skin sensitivity should be normal, which were evaluated by means of the* Mini Mental State Examination* [20] and* Pain and Light Touch Sensation* tests [21], respectively. All the stroke patients were independently evaluated by two experienced physical therapists. Individual characteristics of all stroke patients are summarized in Table 1.

The entire experimental protocols were submitted to the local ethical committee (process number: 082/08) and were conducted after each volunteer gave informed consent.

### 2.2. Experimental Designs

The first and the second groups (Experiments I and II) were subjected to four different frequencies of SES: 3, 30, 150, and 300 Hz. The frequencies were chosen based on previous reports in which they resulted in motor control improvement and/or neuromodulation [2, 22–24] by using SES and other therapeutic modalities of electrical stimulation. The frequencies of stimulation were applied in a random manner in both experiments. In Experiment I, the effects of the four frequencies of SES upon corticospinal and spinal excitability were evaluated in the same experimental session. Each frequency of SES was applied for 5′ and was followed by a rest interval of 10′. Motor evoked potentials (MEPs) and H-reflex were collected immediately before (baseline) and after (0′) SES application and then 5′ later (5′). In contrast, in Experiment II, the volunteers were subjected to 30′ of SES for each frequency and the protocol was conducted on four different days separated by a minimum interval of 72 hours. Similarly to Experiment I, MEPs and H-reflex were collected immediately before (baseline) and after (0′) SES application and then 5′ (5′) and 10′ (10′) later. Each single experimental session lasted for approximately 4 h.

In Experiment III, the stroke patients were submitted to SES therapy only at 3 Hz that was also applied for 30′ in one single session, similarly to Experiment II. We justify such decision due to the time consumption (~4 h) of Experiments I and II and because previous authors [14] report interesting M1 modulatory effects when SES was applied below 10 Hz. Additionally, the passive mechanical resistance of the right wrist joint in the extension movement was evaluated by means of an isokinetic system [25] only before (baseline) and immediately after (0′) SES therapy.

### 2.3. SES Application

**Figure 1:**
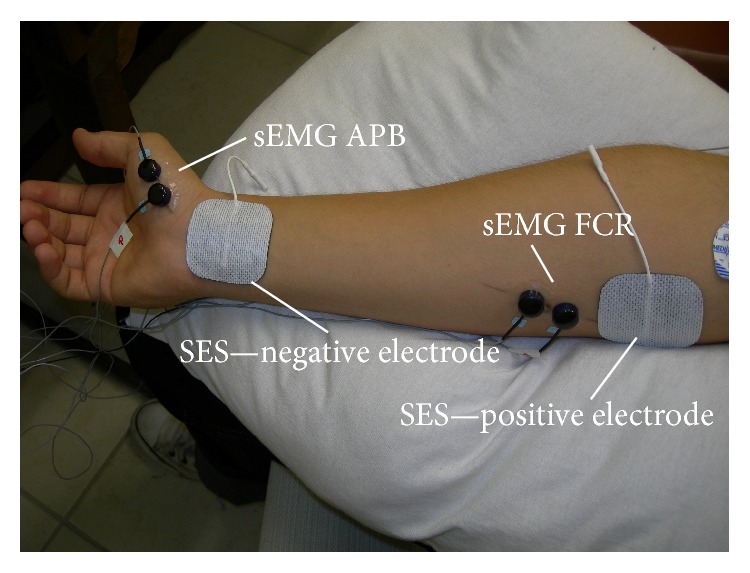
Surface electrode placement for SES. The positive electrode was placed near the elbow joint while the negative electrode was placed near the wrist joint. Surface electromyographic electrodes (in black) can be observed over the* flexor carpi radialis* (FCR) and* abductor pollicis brevis* (APB) muscle bellies.

The SES pulse was a constant amplitude current waveform (an unbalanced asymmetrical biphasic pulse) with duration of 500 *μ*s [26, 27]. SES was delivered using a custom electrical stimulator (FES-PEB) built by Velloso and Souza [27]. Pulse intensity was set below the MT and was determined based on the volunteers' reports of a tingling sensation in the stimulated area (forearm flexor muscles) without any pain or visible movement of the wrist and fingers. Surface self-adhesive electrodes (5 × 5 cm; model: CF5050, Axelgaard Manufacturing Co., Ltd., Denmark) were used for SES. SES intensities were set between 2–7 mA (Experiment I), 3–8 mA (Experiment II), and 3–5 mA (Experiment III). The surface electrodes were positioned over the right forearm, between the wrist (negative electrode) and the elbow (positive electrode) joints, parallel to the FCR muscle in the longitudinal direction (Figure 1).

Because it has been suggested that forearm rotation around its longitudinal axis may modulate spinal reflexes [28], the volunteers remained seated in a comfortable chair with the right forearm maintained in a prone position throughout the experiments.

### 2.4. TMS and H-Reflex Data Acquisition

A BIOPAC system (model: MP150; A/D converter: 16 bits; dynamic range: ±10 V; sampling frequency: 15 kHz; band pass filter: 4th order and 100–5000 Hz; gain: 2000; BIOPAC Systems, Inc., USA) was used to collect the MEPs and H-reflex. Surface BIOPAC reusable electrodes (Ag/AgCl; diameter: 8 mm) were placed following SENIAM recommendations [29] over the following muscle bellies: the* flexor *(FCR) and* extensor* (ECR)* carpi radialis* and* abductor pollicis brevis* (APB). The ECR and APB muscles were defined as controls in Experiments I and II. In Experiment III, we monitored one more muscle: the contralateral* flexor carpi radialis* (FCRc) from the nonaffected left forearm. Therefore, in this particular experiment, the FCRc, ECR, and APB muscles were defined as controls.

A TMS* butterfly* coil (model: MagPro; MagVenture, Denmark) was positioned over the left M1 in the optimal scalp position (*hot-spot*) to elicit FCR motor responses in the contralateral hemibody. The* hot-spot* position was achieved using a cap with marks (grid: 1 × 1 cm) according to the international 10-20 system for electroencephalography. The FCR* hot-spot* was defined as the site in M1 in which a single magnetic pulse set at a minimum intensity produced a MEP response (amplitudes with more than 50 *μ*V in three out of six trials) in the relaxed muscle [30–32]. Then, the magnetic stimulus intensity was adjusted to 20% above the FCR resting MT.

As previously mentioned, MEPs were collected from all the muscles studied immediately before (baseline) and immediately after (0′) SES and up to 5′ (Experiment I) and 10′ (Experiments II and III) later after each session of SES and for each frequency. In each measurement, at least six (Experiment I) and ten (Experiments II and III) single TMS pulses were delivered with an interpulse interval of 5–10 s. The surface electromyographic (sEMG) data acquisition was triggered by a hardware pulse provided by the TMS system. Changes in spinal excitability were monitored through the evaluation of the mean value between the maximum and the minimum FCR H-reflex responses obtained by means of a curve of recruitment collected before the beginning of each SES session. To elicit the H-reflex from the FCR, a rectangular monophasic pulse of 800 *μ*s was applied to a pair of surface electrodes, where one of the electrodes was placed over the median nerve (cathode: Ag/AgCl; 4 mm diameter), ~4 cm above the elbow joint, and the other on the opposite limb (anode: Ag/AgCl; 47.5 cm^2^). The sEMG signal acquisition was started by a hardware pulse trigger provided by the stimulator. Once the electrical pulse was applied, a data window of 100 ms was collected and saved. The interpulse intervals varied between 5 and 10 s [33]. At least four sEMG signal data windows were obtained for further analysis due to the robustness of the collected H-reflex data. Regarding the FCR H-reflex, Experiment I provided suitable data only from seven volunteers (one man and six women; age: 19.0–31.0 years; height: 1.55–1.73 m; body mass: 53.3–82.6 kg; Edinburgh Handedness Inventory [17]: +60.0–+90.0) due to methodological constraints in recording it. Similarly, also due to methodological constraints, it was possible to collect the FCR H-reflex only from two stroke patients and, therefore, we decided not to present these data in this study.

Visual feedback of the sEMG signals was provided throughout all the experimental sessions to certify that all the volunteers were relaxed.

### 2.5. The Passive Wrist Torque Measurement

**Figure 2:**
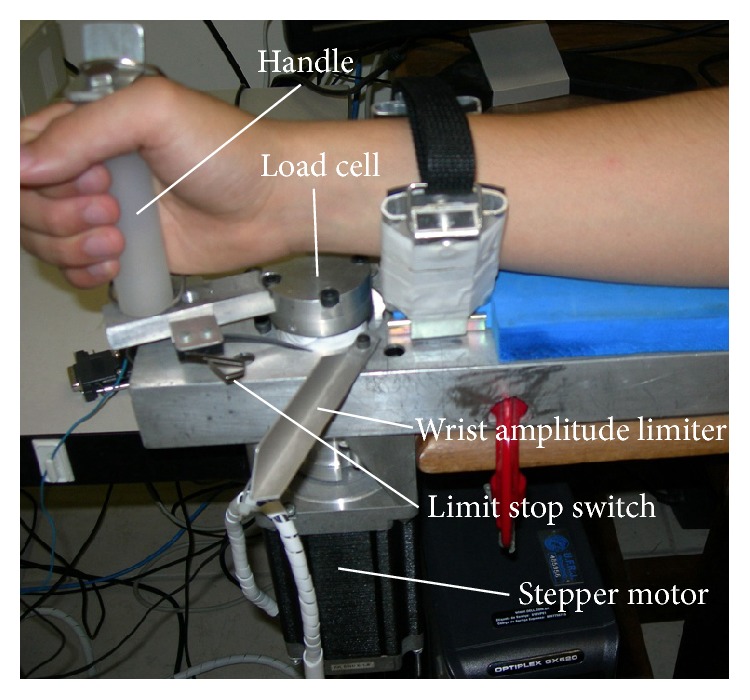
The isokinetic system that was built for measuring the passive mechanical resistance in the wrist joint. It provides the torque and angle data from cyclical wrist extensions with constant angular speed and range of movement.

A custom-made device was built to evaluate the passive resistance of the wrist joint while being moved (Figure 2). It consisted of a stepper motor of 100 kgf·cm static torque controlled by a micro-step driver to make small steps of 0.036° that smoothed the movement. Attached to the stepper motor, a load cell allowed measuring the torque applied to the wrist. The data was recorded using a Spider 8 (HBM, Hottinger Baldwin Messtechnik) system with 16 bits, 4800 Hz sample rate, and an antialiasing filter set at 960 Hz.

The root mean square (RMS) value of the force measured by a load cell was calculated and used as an estimate of the torque (gf·cm) during 5 cycles of 35 degrees of wrist extension set at an angular velocity of 10 degrees per second to avoid the stretch reflex [34].

### 2.6. Data and Statistical Analyses

Surface EMG signals collected from each muscle during Experiments I, II, and III and at each interval of time (baseline, 0′ and 5′ or 10′) were analyzed using an algorithm built in Matlab 6.5 (Mathworks, USA). This algorithm measured MEP and H-reflex peak-to-peak (P-P) values, which represents corticospinal [35] and spinal [36] excitability. MEP and H-reflex median values were then obtained from each six (Experiment I) and ten (Experiments II and III) sEMG signal data windows were from the muscles previously mentioned for each group, resulting in one value for each interval of time, that is, before (baseline) and after (0′) SES application, as well as 5′ (Experiment I) and 10′ (Experiments II and III) later for each SES frequency and for each volunteer. The medians from MEP_P-P_ and H-reflex_P-P_ values were then normalized by computing the ratio between the data for each interval of time (0′, 5′, and 10′) and the corresponding baseline value achieved before SES multiplied by 100 [2, 37–39].

All normalized MEP data values were analyzed using a two-way repeated measures ANOVA (factors:* interval of time* ×* frequency*) for each muscle individually. For the H-reflex data, a two-way repeated measures ANOVA (factors:* interval of time* ×* frequency*) was used, as all measures were taken only from the FCR muscle. For Experiment III (TMS and H-reflex), the analysis was performed using a two-way repeated measures ANOVA (factors:* interval of time* ×* muscle*) while the isokinetic data evaluated by means of Student's paired *t*-test. The level of significance (*α*) was set at 5%. The statistical analysis was performed using R, version 3.1. The results are presented in terms of means and standard deviations.

## 3. Results

### 3.1. Experiment I

**Figure 3:**
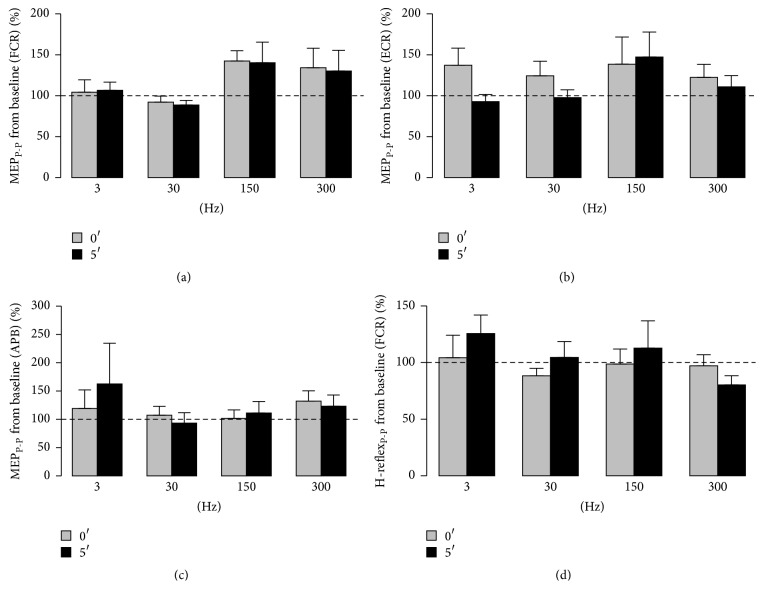
Normalized MEP_P-P_ values collected immediately after (0′) SES and up to 5′ later at the four frequencies (3, 30, 150, and 300 Hz) for the* flexor* (FCR) (a) and* extensor* (ECR) (b)* carpi radialis* and the* abductor pollicis brevis* (APB) (c). (d) depicts the results obtained for normalized FCR H-reflex_P-P_ values immediately after (0′) SES and up to 5′ later for the four frequencies of SES. The dotted line provides a reference from the baseline (before SES) and relative deviations of the results obtained after SES.

Figure 3 depicts the results obtained for normalized MEP_P-P_ and FCR H-reflex_P-P_ values, respectively, immediately after (0′) SES and up to 5′ later for the four frequencies of SES applied during 5′ and for the three muscles (FCR, ECR, and APB). The results obtained after SES set at all the investigated frequencies did not reveal any significant difference among* frequencies* (*P* > 0.155) and* intervals of time* (*P* > 0.087) in MEP_P-P_ data as compared to the baseline for all the muscles (Figures 3(a), 3(b), and 3(c)).

Concerning the normalized FCR H-reflex_P-P_ values (Figure 3(d)), it also did not show any significant difference among* frequencies* (*P* = 0.469) and* intervals of time* (*P* = 0.177) from the baseline.

### 3.2. Experiment II

**Figure 4:**
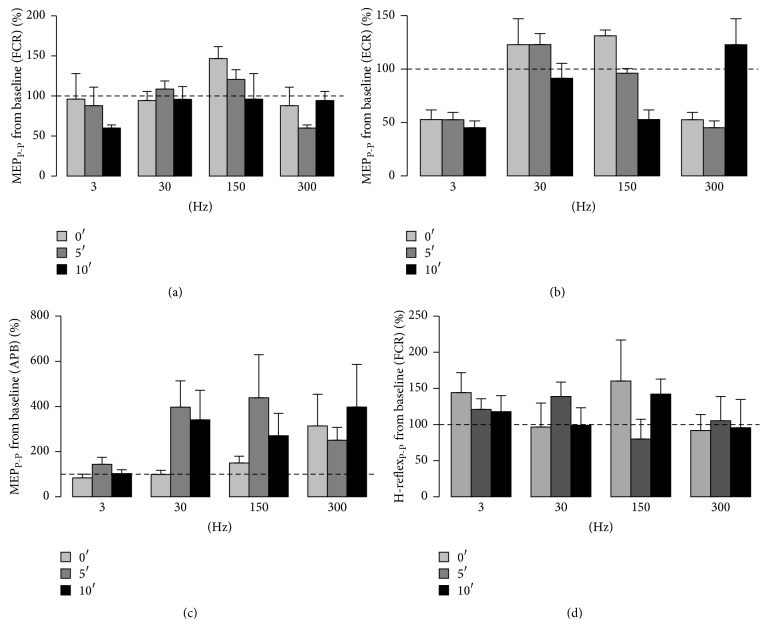
Normalized MEP_P-P_ values collected immediately after (0′) SES and up to 5′ and 10′ later at the four frequencies (3, 30, 150, and 300 Hz) and for the* flexor* (FCR) (a) and* extensor* (ECR) (b)* carpi radialis* and the* abductor pollicis brevis* (APB) (c). (d) depicts the results obtained for normalized FCR H-reflex_P-P_ values immediately after (0′) SES and up to 5′ and 10′ later for the four frequencies of SES. The dotted line provides a reference from the baseline (before SES) and relative deviations of the results obtained after SES.

Figure 4 depicts the results obtained for normalized MEP_P-P_ and FCR H-reflex_P-P_ values, respectively, immediately after (0′) SES and up to 5′ and 10′ later for the four frequencies of SES applied during 30′ and for the three muscles (FCR, ECR, and APB). The results obtained after SES set at all the investigated frequencies did not reveal any significant difference among* frequencies* for FCR (*P* = 0.443) and APB (*P* = 0.524) muscles. Although there was a significant difference among frequencies for ECR (*P* = 0.006), they were not statistically different from baseline (*P* > 0.05). Similarly to Experiment I, there was also no significant difference among* intervals of time* (*P* > 0.235) in MEP_P-P_ data from the baseline for all the muscles (Figures 4(a), 4(b), and 4(c)).

Our results did not also show any significant difference among* frequencies* (*P* = 0.638),* intervals of time* (*P* = 0.563), and interaction of* interval of time* ×* frequency* (*P* = 0.112) concerning normalized FCR H-reflex_P-P_ values from the baseline.

### 3.3. Experiment III

**Figure 5:**
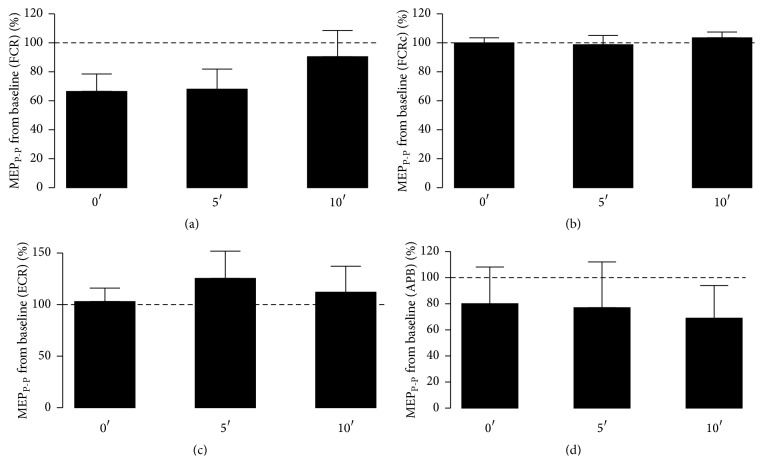
Normalized MEP_P-P_ values collected immediately after (0′) SES and up to 5′ and 10′ later at 3 Hz for the ipsi (FCR (a)) and contralateral (FCRc (b))* flexor carpi radialis*, the* extensor carpi radialis* (ECR), (c) and the* abductor pollicis brevis* (APB) (d). The dotted line provides a reference from the baseline (before SES) and relative deviations of the results obtained after SES.

**Figure 6:**
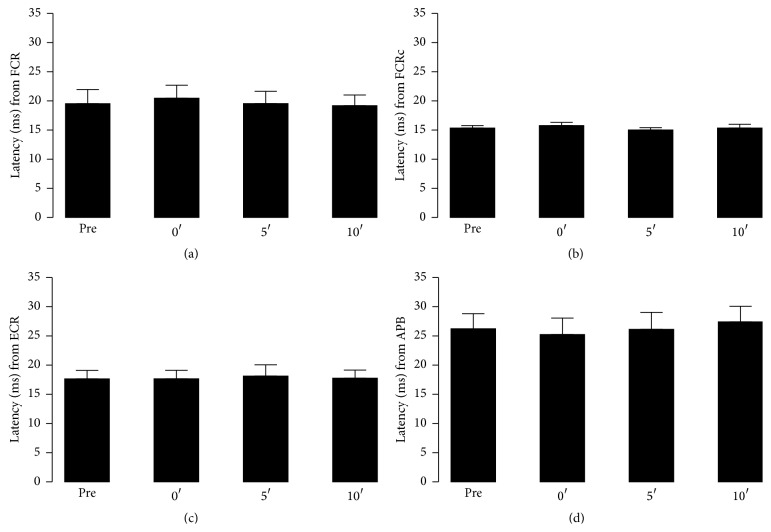
Latency (ms) collected before (Pre) and immediately after (0′) SES and up to 5′ and 10′ later at 3 Hz for the ipsi (FCR (a)) and contralateral (FCRc—nonaffected side (b))* flexor carpi radialis*, the* extensor carpi radialis* (ECR (c)), and the* abductor pollicis brevis* (APB) (d).

Figures 5 and 6 depict the results obtained for the normalized MEP_P-P_ values and latency, respectively, for SES set at 3 Hz and during 30′. There were no significant differences among* intervals of time* in normalized MEP_P-P_ relative values from the baseline for all the three muscles, that is, the FCR (*P* = 0.092), FCRc (*P* = 0.172), ECR (*P* = 0.814), and APB (*P* = 0.864). Latency measures also did not show any significant difference (*P* > 0.150) concerning baseline values.

**Figure 7:**
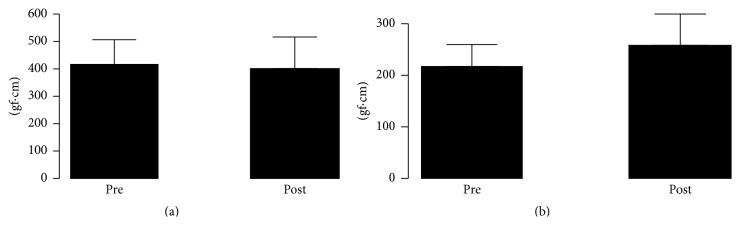
The torque (gf·cm) values from the resistance to the passive extension ((a) *P* = 0.094) and flexion ((b) *P* = 0.774) movements of the wrist joint collected before (Pre) and immediately after (0′) (Post) SES for the five stroke patients diagnosed with spasticity.

The resistance to passive extension (Figure 7(a); *P* = 0.094) and flexion (Figure 7(b); *P* = 0.774) movements of the wrist joint, evaluated by means of the isokinetic system, also did not show any significant difference for the comparison of measurements collected immediately after (0′) (Post) SES and the baseline (Pre).

## 4. Discussion

It has been proposed that the use of SES in the clinical field can provide improvements similar to those obtained with intensive training [40]. For instance, Conforto et al. [41] observed an increase in the force level of pinch movements in stroke patients after two hours of SES. Dos Santos-Fontes et al. [9] also suggested that SES might lead to long-lasting improvements of paretic arm performance in chronic stroke patients. Furthermore, SES has been proposed as an alternative approach in minimizing spasticity [6, 7, 42], although there is also no clarity concerning its underlying mechanisms. Hence, despite the above-mentioned examples concerning the positive effects of SES in a clinical setting, there is still no consensus regarding how some of the parameters used in SES (e.g., application time, frequency, intensity, waveform, and pulse width) are taken into account by the central nervous system (CNS) [14, 43]. Therefore, we decided to evaluate the effect of different frequencies of SES in the spinal and corticospinal excitability of healthy volunteers. In addition, we also performed a pilot study to evaluate the effect of SES applied for 30′, in line with clinical practice, and chose a frequency of 3 Hz applied over the spastic forearm flexor muscles of five stroke patients by means of TMS and passive wrist movement assessment.

Nevertheless, what does the literature tell us about different frequencies of SES in the corticospinal modulation and spasticity? Excitatory as well as inhibitory effects in M1 were shown to be maintained from minutes to hours after SES therapy [4, 13, 37, 44, 45]. Even though some authors have suggested that an increase in the stimulation frequency might lead preferentially to an increase in M1 excitability [13, 22, 38, 46, 47], others reported an opposite effect at the spinal level [48]. We hypothesized that an increase in frequency would allow the ionic current to flow more deeply over the full forearm extent and around the median nerve, which innervates the skin of the palmar side of the thumb, the index, the middle finger, and the APB muscle [49]. As a result, we expected to induce changes in corticospinal excitability of the FCR and APB muscles. However, we also considered the hypothesis of observing some effect in the ECR muscle given that type II afferent fibers (nonadapting sensory fibers) in muscles are able to facilitate or inhibit antagonist muscles [50], although Pierrot-Deseilligny and Burke [36] state that the effect of this interaction on the upper limb is not well understood.

Nevertheless, in the present study, both short (5′) and long (30′) durations of SES applied at different frequencies did not lead to changes in spinal and corticospinal excitability of the muscle under stimulation nor at the muscles that share any kinesiologic property with the stimulated one. Despite the discouraging results, some issues in addition to the frequency of stimulation must be taken in account and they are discussed as follows.

### 4.1. Pulse Width

We used a longer pulse width (500 *μ*s) than have other authors (100 to 300 *μ*s) [2, 6, 22], which are more selective [26]. We decided on this pulse width due to the need of recruiting a wide range of diameters and modalities of sensory afferents fibers. As our main aim was to evaluate the effect of different frequencies of SES, an increase in the selectivity of recruitment could mask a possible source of a corticospinal excitability modulation derived from this parameter only.

Similar negative and divergent results to ours have also been reported in the literature. For instance, Tinazzi et al. [2] observed an increase and a decrease in the corticospinal excitability of ECR and FCR, respectively, after applying SES at 150 Hz over the FCR muscle for 30′. On the other hand, Fernandez-del-Olmo et al. [49] performed a similar protocol and did not observe any facilitation or inhibition of both muscles. Curiously, both studies used a narrower pulse (100 *μ*s), which is expected to be more selective for larger diameter fibers [16].

A shorter pulse width is expected to achieve higher selectivity in discriminating between somatosensory, motor, and pain sensory nerves. A pulse width of 500 *μ*s may result in a greater summation of responses from receptors distributed close to the site of SES, which will be integrated and processed at different levels of the CNS and likely transmitted to M1 as an “averaged” signal. Thus, as a first hypothesis to explain the lack of SES modulation we suggest that this averaged input might not be able to induce any spinal or corticospinal modulation.

### 4.2. The Intensity of SES Stimulation

Another important issue concerns the intensity of the stimulation. Some studies report divergent effects of SES set at different intensity levels in corticospinal excitability but also including paired associated stimulation (PAS) protocols. In this context, Pitcher et al. [22] applied electrical stimulation above the MT and set at 3 Hz over the* first dorsal interosseous* (FDI) muscle during 30′ while pulses of TMS were synchronized over the FDI* hot*-*spot* in a PAS protocol. They observed a decrease in MEP_P-P_ for approximately 40–50′, suggesting that peripheral electrical stimulation at such low frequency mediates the recruitment of neural circuits that induce long-term depression (LTD). Alternatively, Aimonetti and Nielsen [51] showed that applying a conditioning electrical stimulus just below the MT over the median nerve, which supplies the FCR, produced a facilitation of the ECR for a very short term. In contrast, Bertolasi et al. [52] also showed that applying a conditioning electrical stimulus just above the MT over the median nerve produced an ECR inhibition. Veldman et al. [14] considered that the corticospinal excitability level appears to be modulated by a fine-tuning of SES intensity, which can vary from the perceptual to the motor threshold and be explained by the wide range and directions of neuronal responses obtained from the MEPs in TMS experiments. Moreover, their findings suggest that a stimulation intensity set at the perceptual threshold would not be capable of inducing any modulation in the corticospinal excitability. Based on these remarks and on the methodological approach adopted in the present study to tune the SES intensity (see details above in Section 2), as a second hypothesis we conjecture that, being provided at or very near the perceptual threshold for most of the volunteers, SES did not induce any measurable physiological effect for all the tested muscles, frequencies, and intervals of time.

### 4.3. The Maintenance of SES Intensity

Another important aspect concerns the maintenance of the stimulation intensity. Different authors adjust the intensity of SES during their experiments to avoid habituation [3, 41, 53, 54]. Unlike most of authors, we decided to maintain the same stimulation intensity to avoid a* bias* in the evaluation of corticospinal modulation after SES. Moreover, we evaluated two different application times of SES: a very short (5′) and a long (30′) one. Therefore, habituation mechanisms must be taken into account even in the SES protocol of short duration. In line with this third hypothesis, the works of Dobkin [55] and Dimitrijević et al. [56] supported the idea that a regular and constant pattern of SES can lead either to habituation/accommodation very quickly or to a failure in producing any change in M1 excitability, although this process does not seem to be rigid. Thus, we suggest as a third hypothesis that a habituation effect induced by the maintenance of the stimulation intensity could be an additional variable of not inducing any spinal or corticospinal modulation from SES in the present study.

### 4.4. The H-Reflexes_P-P_ Evaluation

Even though we have faced methodological constraints to record the H-reflex from some healthy volunteers, SES seemed to fail to induce changes in the excitability of such measurement in FCR in Experiments I and II. Despite the fact that the H-reflex is generally considered a monosynaptic response, it may be also modulated by other inputs which converge to common interneurons and that may receive inputs from spinal and supraspinal sources [7, 15]. Therefore, SES seems to stimulate differently specific receptors which might cancel themselves reciprocally and produce a mixture of excitation and inhibition [7], which was previously assumed as one of our hypotheses of not observing any significant difference in corticospinal excitability.

Departing from the herein shown lack of SES effect upon the FCR H-reflex, some studies [57] have shown that functional electrical stimulation (FES), which allows recruiting Ia muscle spindle sensory neurons, is able to induce modulation of the H-reflex in neurological patients and healthy subjects as well. Thus, it may be suggested that Ia afferents, not likely recruited under the SES protocol adopted in this study, could be able to evoke a FCR H-reflex modulation. Hence, the intensity of stimulation might also be a “key-point” in the modulation of spinal reflexes.

### 4.5. Stroke Spastic Patients

The purpose of this preliminary study was to evaluate for the first time, to our knowledge, the effects of SES set at a low frequency (3 Hz) over the spastic forearm flexor muscles at the corticospinal level in chronic stroke patients. Moreover, we intended to evaluate the carryover effects of SES in the passive mechanical resistance of the impaired wrist joint. Although we did not observe any significant spinal and corticospinal modulation in Experiments I and II, we firstly hypothesized that a decrease in the corticospinal response provided by SES at a low frequency (3 Hz) [22] would be capable of contributing to decreasing temporarily such muscle overactivity. However, despite the negative results, different authors have provided some interesting arguments and results that preliminarily reinforce our previous hypothesis. As already cited, Pitcher et al. [22] observed that peripheral electrical stimulation at a low frequency results in a decrease in corticospinal excitability, although the hypotheses concerning the likely mechanisms under the effects of this electrical stimulation pattern in spasticity are still lacking. Notwithstanding, Liepert et al. [58] suggested that the intracortical inhibition might be reduced in stroke patients, due to a decreased GABAergic (inhibitory) and/or an increased glutamatergic (excitatory) activity, respectively. The unbalance of those neurotransmitters might contribute to the manifestation of spasticity of supraspinal origin although its physiopathology is still under investigation [59]. Likewise, long-term (>30′) SES at low frequencies might be able to conduct to an increase in the recruitment of GABAergic circuitries and conduct to a depression in corticospinal excitability. Pitcher et al. [22] also support that the modulation of those neural circuitries may be frequency-dependent and might be optimized by 3 Hz stimulation. Sonde et al. [60] also investigated SES set at a low frequency (1.7 Hz) in the treatment of spasticity in individuals with stroke and despite the improvements in the motor pattern, they did not also observe any significant decrease in spasticity, as evaluated by means of the Ashworth scale.

Even though the MEPs and wrist torque data did not reach statistically significant levels, the evaluation of the mechanical resistance by means of an isokinetic system, that is, a more accurate and sensible approach, four out of six patients presented relative decreases (5.5 to 14.3%) in passive wrist torque (flexion and extension), which might be seen as clinically significant. It is important to highlight that the Ashworth scale does not provide sensitivity to minimal variations on the level of spasticity.

A hyperexcitability of H-reflex has been considered as an index of spasticity [48, 61]. Therefore, the lack of change in H-reflex measurements does not allow implying spinal instead of corticospinal excitability as the likely portion of the CNS suitable to SES effects in stroke patients with spasticity.

In summary, as we expected to observe a covariation between the MEP behavior with a decrease in resistance to the passive extension movement of the wrist joint, we may also assume that a low frequency of SES set at 3 Hz and the perceptual threshold might be unable to induce any neuromodulation in these patients.

## 5. Conclusion

Based on the work of Ward [16], we proposed that an increase in the frequency of SES stimulation would allow the ionic current to flow more deeply and so a larger pool of somatosensory receptors from different tissues adjacent to the SES would be recruited. However, even though we must recognize small participant size samples in Experiments II and III, the results provided by our experiments suggest that none of the investigated frequencies (3, 30, 150, and 300 Hz) of SES along with all the other chosen parameters seem to be able to operate as a* key* in switching modulatory effects in the CNS of healthy volunteers and stroke patients with spasticity.

## Figures and Tables

**Table 1:** Main characteristics of the stroke patients.

Patient	Age	Sex	Months after stroke	Prestroke hand dominant hemisphere	Lesioned hemisphere	Lesion type	Modified Ashworth scale
P1	59	M	37	Left	Left	Ischemic	2.0
P2	69	M	18	Left	Left	Ischemic	1.0
P3	45	M	48	Left	Left	Hemorrhagic	2.0
P4	63	F	24	Left	Left	Ischemic	1.0+
P5	70	M	51	Left	Left	Hemorrhagic	2.0
